# Supplementary material for: Eye-head coordination during goal-directed orienting in mice
Source: Commun Biol. 2026 Apr 3;9:732. doi: 10.1038/s42003-026-09943-x (PMC13219551; doi:10.1038/s42003-026-09943-x)
Supplement: Supplementary file 2 — Description of Additional Supplementary Files [file 42003_2026_9943_MOESM2_ESM.pdf]

Description of Additional Supplementary Files

File name: Supplementary Video 1

Description: Eye and head position during Active: Head-initiated gaze redirection, Related to Figure 2, Figure 6, Figure S1

File name: Supplementary Video 2

Description: Eye and head position during Active: Eye-Head Co-Initiated gaze redirection, Related to Figure 2, Figure 6, Figure S1

File name: Supplementary Video 3

Description: Eye and head position during Passive VOR, Related to Figure 6

File name: Supplementary Video 4

Description: Eye and head position during Passive with Quick-Phase, Related to Figure 6
