# Supplementary material for: Eye-head coordination during goal-directed orienting in mice
Source: Commun Biol. 2026 Apr 3;9:732. doi: 10.1038/s42003-026-09943-x (PMC13219551; doi:10.1038/s42003-026-09943-x)
Supplement: Supplementary file 7 — Reporting Summary [file 42003_2026_9943_MOESM7_ESM.pdf]

Corresponding author(s): Kathleen Cullen

Last updated by author(s): Mar 17, 2026

Reporting Summary

Nature Portfolio wishes to improve the reproducibility of the work that we publish. This form provides structure for consistency and transparency in reporting. For further information on Nature Portfolio policies, see our Editorial Policies and the Editorial Policy Checklist.

Statistics

For all statistical analyses, confirm that the following items are present in the figure legend, table legend, main text, or Methods section.

n/a	Confirmed	
☐	☒	The exact sample size (*n*) for each experimental group/condition, given as a discrete number and unit of measurement
☐	☒	A statement on whether measurements were taken from distinct samples or whether the same sample was measured repeatedly
☐	☒	The statistical test(s) used AND whether they are one- or two-sided<br>*Only common tests should be described solely by name; describe more complex techniques in the Methods section.*
☒	☐	A description of all covariates tested
☐	☒	A description of any assumptions or corrections, such as tests of normality and adjustment for multiple comparisons
☐	☒	A full description of the statistical parameters including central tendency (e.g. means) or other basic estimates (e.g. regression coefficient) AND variation (e.g. standard deviation) or associated estimates of uncertainty (e.g. confidence intervals)
☐	☒	For null hypothesis testing, the test statistic (e.g. *F*, *t*, *r*) with confidence intervals, effect sizes, degrees of freedom and *P* value noted<br>*Give P values as exact values whenever suitable.*
☒	☐	For Bayesian analysis, information on the choice of priors and Markov chain Monte Carlo settings
☒	☐	For hierarchical and complex designs, identification of the appropriate level for tests and full reporting of outcomes
☐	☒	Estimates of effect sizes (e.g. Cohen's *d*, Pearson's *r*), indicating how they were calculated

Our web collection on statistics for biologists contains articles on many of the points above.

Software and code

Policy information about availability of computer code

Data collection	Pupil positional data was obtained using a custom video oculography code. Head position was monitored in real time using a QNX-based data acquisition system (REX) and SpikeGLX (Janelia).
Data analysis	Data analysis was performed in Matlab 2023b. Statistical analyses were performed in Matlab 2023b and Graphpad Prism 9.

For manuscripts utilizing custom algorithms or software that are central to the research but not yet described in published literature, software must be made available to editors and reviewers. We strongly encourage code deposition in a community repository (e.g. GitHub). See the Nature Portfolio guidelines for submitting code & software for further information.

Data

Policy information about availability of data

All manuscripts must include a data availability statement. This statement should provide the following information, where applicable:

- Accession codes, unique identifiers, or web links for publicly available datasets
- A description of any restrictions on data availability
- For clinical datasets or third party data, please ensure that the statement adheres to our policy

This study did not generate any unique reagents. Any additional information required to reanalyze the data reported in this paper is available from the lead contact upon request. Requests for further information should be directed to the lead contact, Kathleen Cullen, kathleen.cullen@jhu.edu.

Research involving human participants, their data, or biological material

Policy information about studies with human participants or human data. See also policy information about sex, gender (identity/presentation), and sexual orientation and race, ethnicity and racism.

Reporting on sex and gender	N/A
Reporting on race, ethnicity, or other socially relevant groupings	N/A
Population characteristics	N/A
Recruitment	N/A
Ethics oversight	N/A

Note that full information on the approval of the study protocol must also be provided in the manuscript.

Field-specific reporting

Please select the one below that is the best fit for your research. If you are not sure, read the appropriate sections before making your selection.

☒ Life sciences ☐ Behavioural & social sciences ☐ Ecological, evolutionary & environmental sciences

For a reference copy of the document with all sections, see nature.com/documents/nr-reporting-summary-flat.pdf

Life sciences study design

All studies must disclose on these points even when the disclosure is negative.

Sample size	Nine mice were used in this study, comparable to similar studies in the field. Each animal contributed multiple measured trials for subsequent analysis.
Data exclusions	Trials were excluded if: 1) tracking quality was insufficient during the trial; 2) movement execution was incomplete as described in the methods; and 3) eye or head movements did not reach the 25°/s onset threshold as described in the methods. All inclusion/exclusion procedures were automated where possible and applied uniformly across animals and conditions.
Replication	All experiments were replicated across nine C57BL/6J mice trained under identical active and passive conditions, with multiple head and eye movement trials collected per animal.. Calibration and analysis procedures—including VOR gain verification, pupil-to-degree conversion, and automated MATLAB-based signal processing—were standardized to ensure reproducibility and minimize experimenter bias.
Randomization	This study used a within-subject (repeated-measures) design in which all mice of identical genotype served as their own controls, performing both active and passive paradigms on the same apparatus.
Blinding	Blinding is not relevant for this study, as all mice were of the same strain/substrain (C57BL/6J) and served as their own controls.

Reporting for specific materials, systems and methods

We require information from authors about some types of materials, experimental systems and methods used in many studies. Here, indicate whether each material, system or method listed is relevant to your study. If you are not sure if a list item applies to your research, read the appropriate section before selecting a response.

Materials & experimental systems

n/a	Involved in the study	
☒	☐	Antibodies
☒	☐	Eukaryotic cell lines
☒	☐	Palaeontology and archaeology
☐	☒	Animals and other organisms
☒	☐	Clinical data
☒	☐	Dual use research of concern
☒	☐	Plants

Methods

n/a	Involved in the study	
☒	☐	ChIP-seq
☒	☐	Flow cytometry
☒	☐	MRI-based neuroimaging

Animals and other research organisms

Policy information about studies involving animals; ARRIVE guidelines recommended for reporting animal research, and Sex and Gender in Research

Laboratory animals	Adult (6-8 months) male C57bl/6J mice were used in this study.
Wild animals	No wild animals are used in this study.
Reporting on sex	Because the study aimed to characterize fundamental features of mouse eye–head coordination rather than sex-specific differences, the use of a single sex was considered appropriate for this initial investigation.
Field-collected samples	No field collected samples were used in this study.
Ethics oversight	All animal husbandry and experimental procedures were performed in accordance with the National Institutes of Health Guide for the Care and Use of Laboratory Animals and were approved by the Johns Hopkins University Animal Care and Use Committee (ACUC).

Note that full information on the approval of the study protocol must also be provided in the manuscript.

Plants

Seed stocks	N/A
Novel plant genotypes	N/A
Authentication	N/A
